# PhytoKeys at 100: progress in sustainability, innovation, and speed to enhance publication in plant systematics

**DOI:** 10.3897/phytokeys.100.27591

**Published:** 2018-06-21

**Authors:** W. John Kress, Sandra Knapp, Pavel Stoev, Lyubomir Penev

**Affiliations:** 1 National Museum of Natural History, Smithsonian Institution, Washington DC, USA; 2 Department of Life Sciences, The Natural History Museum, London, United Kingdom; 3 National Museum of Natural History, Bulgarian Academy of Sciences, Sofia, Bulgaria; 4 Pensoft Publishers, Sofia, Bulgaria; 5 Institute of Biodiversity and Ecosystem Research, Bulgarian Academy of Sciences, Sofia, Bulgaria

Eight years have passed since the launch of PhytoKeys (Penev et al. 2010) – Pensoft’s flagship journal in plant systematics – and six years from our last editorial commemorating the second year of its existence (Kress et al. 2012). Today we are publishing the journal’s 100th issue! There is no better occasion to look back and consider the development and most significant achievements of PhytoKeys.

**Figure 1.**
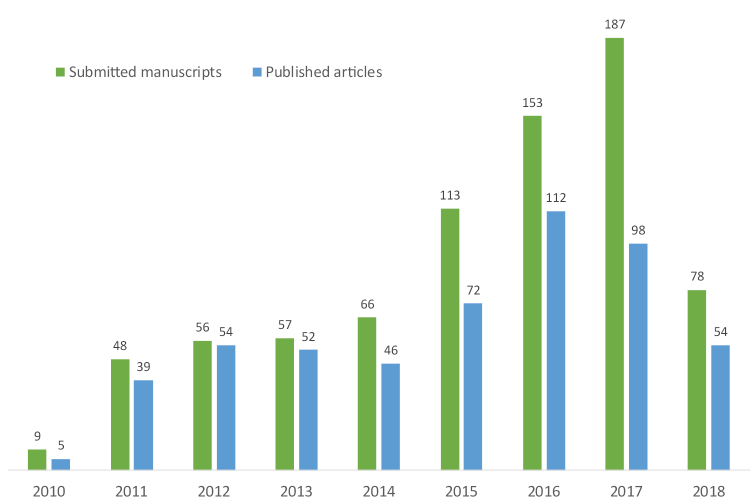
Growth of submitted manuscripts and published articles in PhytoKeys from 2010 to 2018 (until 1.6.2018).

In a very short time period after its inception, PhytoKeys became one of the most popular and appreciated Open Access journals in botany. The journal started with only 48 submissions in 2011; by 2017 that number quadrupled to 187 manuscripts submitted annually (Table 1, Fig. 1). The number of published articles has grown as well, from 39 in 2011 to 112 in 2016, while the number of published pages increased from 75 in 2010 to 3141 in 2016. To date the journal has received in total 759 submissions and published 532 articles, of which 21 are full monographs. The average acceptance rate for the period 2011–2017 was 70%, which we believe is optimal and sustainable for a taxonomic journal.

**Table 1.** Total number of submitted manuscripts, published articles, and printed pages since 2010.

**Year**	**Submitted manuscripts**	**Published articles**	**Published pages**
2010	9	5	75
2011	48	39	397
2012	56	54	1042
2013	57	52	1494
2014	66	46	1342
2015	113	72	2035
2016	153	112	3141
2017	187	98	1973
2018*	70	54	1097
**Total**	**759**	**532**	**12569**

* Until 1.6.2018.

The journal indexes all nomenclatural changes and additions in the International Plant Names Index (IPNI) (Penev et al. 2010, 2016). In all, one new tribe, 26 new genera or subgenera, and 439 new species or infraspecies have been described in the journal since its launch; this equates to 466 new taxa in total. In addition to new taxa, more than 400 new combinations, replacement names, new status designations, and other nomenclatural acts have been proposed in the journal since we began.

Over the years PhytoKeys has attracted a diverse range of botanical researchers from all parts of the world, with the highest numbers coming from the United States of America (193), Brazil (93), China (80), United Kingdom (53) and Germany (49). Altogether 939 scientists from 67 countries have published in the journal from its launch until 1 June 2018 (Table 2).

**Table 2.** Total number of PhytoKeys authors per country.

**Country**	**N**	**Country**	**N**	**Country**	**N**
United States of America	193	Philippines	8	Taiwan	2
Brazil	93	Austria	7	Tanzania	2
China	80	Peru	7	Ireland	2
United Kingdom	53	Slovakia	6	Norway	2
Germany	49	Portugal	6	Mauritius	2
Belgium	39	Czech Republic	6	French Polynesia	2
Turkey	33	Ecuador	5	Hong Kong	1
Australia	27	Denmark	5	French Guyana	1
India	25	Sweden	5	Gabon	1
Netherlands	24	Poland	5	Ukraine	1
Spain	23	Korea, South	5	Uganda	1
France	22	Colombia	4	Cuba	1
Japan	19	Switzerland	4	Uruguay	1
New Zealand	17	Panama	4	Nepal	1
Vietnam	17	Paraguay	3	Lao PDR	1
South Africa	16	Cameroon	3	Uzbekistan	1
Thailand	14	Finland	3	Cambodia	1
Argentina	13	Myanmar	3	Kyrgyzstan	1
Canada	12	Papua New Guinea	3	Kenya	1
Mexico	12	Bulgaria	3	Hungary	1
Russia	10	Singapore	3	Costa Rica	1
Italy	10	Venezuela	3		
Malaysia	8	Chile	2		

In 2015 PhytoKeys was granted its first impact factor of 0.68, and it has gradually increased in the subsequent two years and reached 1.11 in 2017. The increase can be best explained by the stringent peer review of content, improved quality control, and manuscript management. In 2014 the journal was also accepted for coverage by Scopus. In December 2016 Scopus announced the introduction of CiteScore – a new journal level metrics. Currently for 2017, the Cite Score value of PhytoKeys is 1.08.

Along with our overall editorial improvements and advancements, a number of new technological solutions and features have been implemented in PhytoKeys in order to facilitate the efforts of editors, reviewers and authors (see Table 3).

**Table 3.** New technological solutions implemented in the journal.

**Feature**	**For the benefit of**	**Link**	**Use**
Automatic registrations of reviews at Publons	Reviewers and Editors	https://publons.com	Publons helps reviewers and editors get recognition of every review they make for the journal.
Dimensions	Authors, editors, administrators, publisher	https://www.dimensions.ai	Powerful tracker of citations; provides ranking of given research in a given field
Scopus CiteScore Metrics	Authors, editors, administrators, publisher	https://www.scopus.com/sourceid/19700201507	Interactive tool providing information on journal’s performance
Export of published figures & supplementary materials to Biodiversity Literature Repository at ZENODO	Authors, data scientists, community in general	https://zenodo.org/communities/biosyslit/?page=1&size=20	Increases visibility and traceability of article and sub-article elements
Hypothes.is	Authors, readers	http://hypothes.is	Annotations on selected texts from the published article

PhytoKeys content is integrated with a significant number of global indexers and archives, such as PubMedCentral, CLOCKSS, Google Scholar, CAB Abstracts, DOAJ, Vifabio, BHL Citebank, to name just a few. In the two years from 2015 to 2017 Pensoft journals have been integrated with a number of global archives and data repositories that significantly increase visibility and searchability of published content. All journals operating on Pensoft’s innovative platform ARPHA, including PhytoKeys, have benefited from these developments. The list of the online libraries and databases which harvest and manage PhytoKeys content includes:

Library of Congress (USA)

CNKI (China)

CINIPIEC (China)

eLibrary (Russia)

ORCID (International)

Dryad Data Repository (International)

Open Citations Corpus (International)

**Figure 2.**
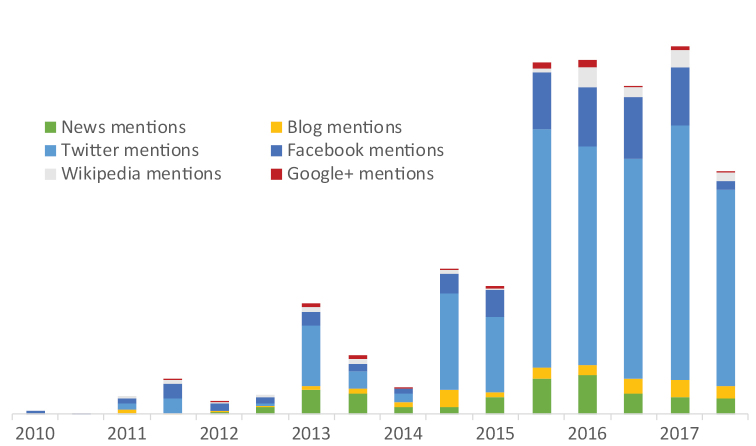
Total number of PhytoKeys mentions in social media and popular magazines.

Since 2016 PhytoKeys has been using Altmetric – a technology providing article level metrics which enables authors to track the online shares and discussions of their published articles. Figure 2 demonstrates the combined results of the social media presence of PhytoKeys articles on Altmetric. The graph clearly shows an increase in the presence and visibility of the published content in social media and popular outlets since September 2016.

Pensoft continues to invest in the popularization of PhytoKeys via media campaigns. Some examples of press releases on articles published in the journal that grasped the attention of journalists and received large media coverage are listed in Table 4. Altogether the top ten articles with the highest number of unique views on PhytoKeys site have received 147,865 views. Four species described in PhytoKeys – the flowering tree named as a new genus *Sirdavidia solannona*, the dragon tree *Dracaena kaweesakii*, the orchid *Telipogon diabolicus* and the bush tomato from northwestern Australia, *Solanum ossicruentum* made it to the top 10 new species nominated by the State University of New York College of Environmental Science’s International Institute for Species Exploration (IISE) (Deutsche Welle, Daily Mail, Publico, CoolEarth, EurekAlert!).

**Table 4.** The top ten PhytoKeys papers that attracted largest media interest.

**Article**	**Press release**	**Media coverage**
Schuette et al. (2018) The hidden *Heuchera*: How science Twitter uncovered a globally imperiled species in Pennsylvania, USA.	Science and Twitter join forces to uncover a globally imperiled plant species	Sverige Radio, Earth.com, PLOS Ecology, IFLScience
Caraballo-Ortiz and Trejo-Torres (2017) Two new endemic tree species from Puerto Rico: *Pisonia horneae* and *Pisonia roqueae* (Nyctaginaceae).	Two Caribbean bird-catcher trees named after 2 women with overlooked botanical works	Der Standard, Mongabay
Diazgranados and Sánchez (2017) *Espeletia praesidentis*, a new species of Espeletiinae (Millerieae, Asteraceae) from northeastern Colombia.	New Colombian plant discovered by Kew scientist honors Colombian president	Express, El Tiempo, La Nacion
Kolanowska et al. (2016) *Telipogon diabolicus* (Orchidaceae, Oncidiinae), a new species from southern Colombia.	Orchid or demon: Flower of a new species of orchid looks like a devil’s head	The Washington Post, FOX news, РИА Новости, El Mundo
Martine et al. (2016) New functionally dioecious bush tomato from northwestern Australia, *Solanum ossicruentum*, may utilize “trample burr” dispersal.	Curious new bush species growing ‘bleeding’ fruits named by a US class of 150 7th graders	Science News, AOL, ABC
Martine et al. (2016) *Solanum watneyi*, a new bush tomato species from the Northern Territory, Australia named for Mark Watney of the book and film “The Martian”.	New bush tomato species is the link between botany and an Oscar-nominated Hollywood movie	Live Science, New York Daily News, Huffington Post
Leopardi-Verde et al. (2016) *Encyclia inopinata* (Orchidaceae, Laeliinae) a new species from Mexico.	Serendipitous orchid: An unexpected species discovered in Mexican deciduous forests	Scientific American, National Geographic Indonesia, Газета.ru
Suetsugu and Fukunaga (2016) *Lecanorchis tabugawaensis* (Orchidaceae, Vanilloideae), a new mycoheterotrophic plant from Yakushima Island, Japan.	Plants cheat too: A new species of fungus-parasitizing orchid	Asian Scientist, Nature World News, La Vanguardia
Couvreur TLP, Niangadouma R, Sonké B, Sauquet H (2015) Sirdavidia, an extraordinary new genus of Annonaceae from Gabon. PhytoKeys 46: 1-19.	A rare new plant inspires the first plant genus named after Sir David Attenborough	The Guardian, Los Angeles Times, Discover Magazine
Fernando E, Quimado M, Doronila A (2014) Rinorea niccolifera (Violaceae), a new, nickel-hyperaccumulating species from Luzon Island, Philippines. PhytoKeys 37: 1-13.	New species of metal-eating plant discovered in the Philippines	International Business Times, Russia Today, Asian Scientist

Over the eight years of the existence of PhytoKeys, the journal has positioned itself among the world’s leading journals in systematic botany. Started by the editors primarily as a taxonomically-oriented journal, the journal has since extended its scope to enable publications across other botanical disciplines, such as plant ecology, genomics, evolutionary biology, paleontology, bioinformatics, ethnobotany, etc.

As the chief editors of PhytoKeys we have worked hard to expand the journal’s editorial board, which has grown significantly and today is comprised of more than 80 experts from various scientific disciplines and geographical areas. The journal has achieved an international reputation by publishing milestone works that will affect all botanists, such as the changes to publication requirements made at the XVIII International Botanical Congress in Melbourne (Knapp et al. 2011a, b), the report on the nomenclature section of the 2005 XVII International Botanical Congress, Vienna (Flann et al. 2015) and the Shenzhen Declaration on Plant Sciences endorsed by 7,000 plant scientists from 77 countries at the XIX International Botanical Congress held in Shenzhen, China (Kress and Knapp 2017).

With its continuous technological innovation and support from subject editors and reviewers, PhytoKeys continues to receive recognition by the international community of plant researchers. This success would not have been possible without our authors, reviewers, subject editors, production staff, readers, and supporters, to which we express our sincerest gratitude and thanks! We cannot wait to see what the 200th issue will look like!
